# Supplementary material for: Community health extension workers’ training and supervision in Ethiopia: Exploring impact and implementation challenges for non-communicable disease service delivery
Source: PLOS Glob Public Health. 2022 Nov 9;2(11):e0001160. doi: 10.1371/journal.pgph.0001160 (PMC10021836; doi:10.1371/journal.pgph.0001160)
Supplement: S1 File — (DOCX) [file pgph.0001160.s001.docx]

**I. Participant information statement and consent form**

1. **What is the research study about?**

The research study aims to explore the system approach to understanding and improving the delivery of Primary Health Care in Ethiopia. You have been invited because you are part of the policy makers/program coordinator/primary health care worker in Ethiopia and your contact was obtained from the Ministry of Health/regional health bureau/woreda(district) health office/your health facility.

1. **Who is conducting this research?**

The study is being carried out by *The George Institute for Global Health, Faculty of Medicine, University of New South Wales, Sydney staff members.*

**Research Funder:** This research is being funded by The George Institute for Global Health, Australia with a seed grant to support research for Under-served community in low-middle Income countries.

1. **Inclusion/Exclusion Criteria**

Before you decide to participate in this research study, we need to ensure that it is ok for you to take part. The research study is looking to recruit people who meet any of the following criteria:

- You are one of the policy makers on health related by the virtue of your position
- You are currently in one of this categories; representatives from the Health extension and Primary Health Service Directorate (Federal MOH)/ national and regional HEP coordinators/ regional Health promotion and diseases prevention team leaders/ programme manager/ HEP supervisor/health care worker

1. **Do I have to take part in this research study?**

Participation in this research study is voluntary. If you do not want to take part, you do not have to. If you decide to take part and later change your mind, you are free to withdraw from the study at any stage.

If you decide you want to take part in the research study, you will be asked to:

- Read the information carefully (ask questions if necessary);
- Sign and return the consent form if you decide to participate in the study;
- Take a copy of this form with you to keep.

1. **What does participation in this research require, and are there any risks involved?**

Participation in an interview

- If you decide to take part in the research study, you will be asked to participate in a face to face interview. You will be asked questions about the overall process and the challenges faced by policy makers in providing health care services for non-communicable diseases at the level of the community. It should take approximately **45-60 minutes** to complete.
- To ensure we collect the responses accurately, we seek your permission to digitally record the interview using an audio tape. If you would like to participate but do not wish to be recorded, you will need to discuss the options for your participation with the research team. For this, the research team we will ask you to sign the consent form and then will continue with the questions. This process will take approximately **10-15 minutes** to complete.

1. **What are the possible benefits to participation?**

We hope to use information we get from this research study to benefit others who seek to understand a system approach to delivering NCD services through non-physician health workers at the primary health care level. This can also help in addressing challenges faced by the health workers and the system at this level.

1. **What will happen to information about me?**

By signing the consent form, you consent to the research team collecting and using information about you for the research study. Your data will be kept for a minimum of 5 years after the publication of the outcome of this research or after the project’s completion. We will store information about you in a non-identifiable format at the George Institute for Global Health office in Sydney, Australia. Your information will only be used for this research study only. Although the results of this research study will be published and/or presented in a variety of forums but in any publication and/or presentation, information will be published in a way such that you will not be individually identifiable.

The information you provide is personal information for the purposes of the Privacy and Personal Information Protection Act 1998 (NSW). You have the right of access to personal information held about you by the University, the right to request correction and amendment of it, and the right to make a complaint about a breach of the Information Protection Principles as contained in the PPIP Act. Further information on how the University protects personal information is available in the [**UNSW Privacy Management Plan**](https://www.legal.unsw.edu.au/compliance/privacyhome.html).

1. **How and when will I find out what the results of the research study are?**

The research team intend to publish and/ report the results of the research study in a variety of ways. All information published will be done in a way that will not identify you.

If you would like to receive a copy of the results you can let the research team know by including your details in the space provided in the consent form.

1. **What if I want to withdraw from the research study?**

If you do consent to participate, you may withdraw at any time. You can do so by completing the ‘Withdrawal of Consent Form’ which is provided at the end of this document. Alternatively, you can ring the research team particularly Azeb Tesema and tell them you no longer want to participate. Your decision not to participate or to withdraw from the study will not affect your relationship with UNSW Sydney or The George Institute for Global Health, Australia.

If you decide to leave the research study, the researchers will not collect additional information from you. Any identifiable information about you will be withdrawn from the research project.

1. **What should I do if I have further questions about my involvement in the research study?**

The person you may need to contact will depend on the nature of your query. If you require further information regarding this study or if you have any problems which may be related to your involvement in the study, you can contact the following member/s of the research team:

**Research Team Contact Details**

| **Name** | Azeb G. Tesema |
| --- | --- |
| **Position** | Student investigator |
| **Email** | a.tesema@student.unsw.edu.au |

**What if I have a complaint or any concerns about the research study?**

If you have a complaint regarding any aspect of the study or the way it is being conducted, please contact the UNSW Human Ethics Coordinator: [humanethics@unsw.edu.au](mailto:humanethics@unsw.edu.au)

**Consent Form – Participant providing own consent**

**Declaration by the participant**

- I understand I am being asked to provide consent to participate in this research study;
- I have read the Participant Information Sheet or someone has read it to me in a language that I understand;
- I understand the purposes, study tasks and risks of the research described in the study;
- I understand that the research team will audio record the interviews; I agree to be recorded for this purpose.
- I provide my consent for the information collected about me to be used for the purpose of this research study only.
- I have had an opportunity to ask questions and I am satisfied with the answers I have received;
- I freely agree to participate in this research study as described and understand that I am free to withdraw at any time during the study and withdrawal will not affect my relationship with any of the named organisations and/or research team members;
- I would like to receive a copy of the study results via email or post, I have provided my details below and ask that they be used for this purpose only;

**Name: _____________________________________**

**Address: ___________________________________**

**Email Address: ______________________________**

- I understand that I will be given a signed copy of this document to keep;

**Participant Signature**

| Name of Participant (please print) |  |
| --- | --- |
| Signature of Research Participant |  |
| Date |  |

**Declaration by Researcher***

- I have given a verbal explanation of the research study, its study activities and risks and I believe that the participant has understood that explanation.

**Researcher Signature***

| Name of Researcher (please print) |  |
| --- | --- |
| Signature of Researcher |  |
| Date |  |

**^+^An appropriately qualified member of the research team must provide the explanation of, and information concerning the research study.**

**Note: All parties signing the consent section must date their own signature.**

**Form for Withdrawal of Participation**

I wish to **WITHDRAW** my consent to participate in this research study described above and understand that such withdrawal **WILL NOT** affect my relationship with The University of New South Wales and The George Institute for Global Health, Australia. In withdrawing my consent I would like any information which I have provided for the purpose of this research study withdrawn.

**Participant Signature**

| Name of Participant  (please print) |  |
| --- | --- |
| Signature of Research Participant |  |
| Date |  |

**The section for Withdrawal of Participation should be forwarded to:**

| CI Name: | David Peiris |
| --- | --- |
| Email: | [dpeiris@georgeinstitute.org.au](mailto:dpeiris@georgeinstitute.org.au) |

**II. Interview guides**

**A. Key informant interview guide for policy makers (National and regional participants)**

**The aim of the Interview**

This interview is part of the research study “exploring the role of the primary health care in NCDs prevention and management program in Ethiopia, focusing on health extension program”. This study is conducted by George Institute for Global Health Australia and the University of New South Wales (UNSW), Sydney.

| **S.No** | **Main question/exploratory questions** | **Probing questions/ Tell me more about** |
| --- | --- | --- |
| 1 | Introductory question:  Tell me your experience/opinion about the Ethiopian primary health care (PHC) and health extension program (HEP), in terms of the overall health system structure (federal or policy level, at the lower level) |  |
| 2 | Recruitment:  How do you select HEWs for further training/ level IV training? | Who selects them? How? Any challenge? |
| 3 | Training:   - What is your view about HEW’s level IV training?     What is the plan for continuous refreshment training/hands on experience | - What do you think about; - Quality of the training - Adequacy of the training   The current capacity of the HEWs in delivering the service |
| 4 | Role of the HEWs:  - what is the role of PHC unit in terms of NCDs prevention and management?  What about the role of HEWs in NCDs prevention and management program | - What challenges are anticipated during implementation of NCD services through the HEWs? |
| 5 | Supervision:  Would you please tell us about the HEP supportive supervision process? | - Any policy direction to implement the 2nd generation/NCDs - What is the federal/regional bureau role in the supervision of the HEWs? |
| 6 | Opportunity for advancement:  What are the opportunities for further promotion or professional advancement through the HEP? | Focus person delegated for NCDs at the regional level/woreda level  Is the PHC board members aware about NCDs? |
| 7 | Please tell me about national/region/woreda commitment to equip the primary health care unit in terms of equipment and supply | What protocols, guidelines, and other teaching aid are available for the NCD program?  What is your view in using technology to assist the implementation of the program (e.g m-health) |
| 8 | Would you please tell us the policy direction and implementation around HEWs performance assessment | Who do what? Challenges? |
| 9 | What are the biggest challenges for addressing NCDs through the PHC/HEP in the health system? | What do you recommend? |

**B. In-depth interview guide for woreda(district) health office and health centre participants**

| **S.No** | **Main question/exploratory questions** | **Probing questions** |
| --- | --- | --- |
| **1** | Introductory question:  Please tell me your experience/opinion about the HEP, in terms of the overall health system structure, woreda level implementation  In your opinion, how do you evaluate the readiness of the PHC unit especially the HEP in delivering NCDs services |  |
| 2 | Please tell me the role of the PHC unit for NCD prevention and management (like cardiovascular disease and diabetes)?  Please tell me the role of HEWs in NCDs prevention and management program | - Is there any challenges in implementing NCD program at the PHC level? |
| 3 | How do you select HEWs for further training/ level IV training? | Who selects them? How? Any challenge? |
| 4 | Training:   - What is your view about HEW’s level IV training?     What is the plan at the region/woreda level for continuous refreshment | - What do you think about; - Quality of the training - Adequacy of the training - Ways to integrate NCDs training program into the existing Integrated refreshment training program   The current capacity of the HEWs in delivering the service |
| 5 | Role of the HEWs:  - What is the role of PHC unit (especially health centre and health post) in terms of NCDs prevention and management?  What about the role of HEWs in NCDs prevention and management program (CVD and diabetes prevention and control) | What challenges are anticipated in implementing NCD program with the HEWs? |
| 6 | Supervision:  Would you please tell us about the HEP supportive supervision process? | How do you supervise the HEWs in your cluster?  What are the challenges with the supervision process?  Specially to implement the NCDs service? |
| 7 | Opportunity for advancement:  What are the opportunities for further promotion or professional advancement through the HEP? | How Is the new career advancement works?  What are the challenges to achieving this?  Is there any method to evaluate individual performance of the HEW? |

**C. In-depth interview guide for Health extension workers (HEWs) and Urban Health Extension Professionals (UHE-ps)**

| **S.No** | **Main question/exploratory questions** | **Probing questions** |
| --- | --- | --- |
| 1 | Please tell me your experience as Health extension worker (HEW) |  |
| 2 | What is your specific job related to non-communicable diseases? | Which area of work are you focusing for non-communicable diseases (health education on prevention of risk factors, screening or other?)  Support needed to provide NCDs services?  What kind of service can be provided in your level (follow up, counselling)  For Urban HEW, what is your role in NCDs prevention and management? |
| 3 | What is your opinion on the newly added packages through the 2nd generation HEP, eg non-communicable disease | Do you believe additional package related to non-communicable disease will increase workload? |
| 4 | Training  Would you please tell me about the level IV training? | Any challenges with training that may affect your knowledge and skill to provide non-communicable disease care provision  Note: For UHE-ps, not needed. |
| 5 | -What is your opinion on the supervision process? Is that helpful to get the required knowledge and skill in NCD service provision? | Is there any challenges relating the supportive supervision for your overall performance?  What is your opinion in terms of supervision frequency, the way feedback is given for you?  What about improving your performance and motivation? |
| 6 | Incentives  Can you tell us your experience, related to what motivate/demotivate you to work as health extension works?  What incentives do you think would motivate Health extension workers to retain their job? |  |
| 7 | What are the opportunities for further promotion or professional advancement to you? |  |
| 8 | How do you describe your readiness to provide NCD services? | What do you think will be the biggest challenge? |
| 9 | What are your biggest challenges as a HEW? | What improvement are needed to better deliver the 2nd generation health extension program? Particularly to provide non-communicable diseases services? |
