# Supplementary material for: Community health extension workers’ training and supervision in Ethiopia: Exploring impact and implementation challenges for non-communicable disease service delivery
Source: PLOS Glob Public Health. 2022 Nov 9;2(11):e0001160. doi: 10.1371/journal.pgph.0001160 (PMC10021836; doi:10.1371/journal.pgph.0001160)
Supplement: Attachment — Submitted filename: Response to Reviewers.docx [file pgph.0001160.s003.docx]

28^th^ August 2022

**Author Response Letter: PLOS Global Public Health [PGPH-D-21-00231]**

Dear Dr. Dinesh Neupane/Academic Editor

I am writing on behalf of all authors to submit the revised manuscript on “*The influence of health extension workers’ training and supervision on their delivery of non-communicable disease services in Ethiopia*”. We appreciate the opportunity to respond to the reviewers’ comments. We have prepared a point-by-point response to the editorial and reviewers’ comments below and attached a clean and tracked change manuscript accordingly.

**Journal requirements**

I. *Please update your online Competing Interests statement. If you have no competing interests to declare, please state: “The authors have declared that no competing interests exist.”*

- Thank you for bringing the matter to our attention. We have updated the Competing Interests statement in the revised submission.

II. *Please amend your online detailed Financial Disclosure statement. This is published with the article. It must therefore be completed in full sentences and contain the exact wording you wish to be published. Please state what role the funders took in the study. If the funders had no role in your study, please state: “The funders had no role in study design, data collection and analysis, decision to publish, or preparation of the manuscript.”*

- We have made the required changes in the revised submission.

III. *Please ensure that the funders and grant numbers match between the Financial Disclosure field and the Funding Information tab in your submission form. Note that the funders must be provided in the same order in both places as well.*

- We have revised the Financial Disclosure and the Funding Information files, as suggested in the resubmission form.

IV. Guidelines for resubmitting your figure files are available below the reviewer comments at the end of this letter

- We have checked the figure file using the Preflight Analysis and Conversion Engine (PACE) digital diagnostic tool and now it meets the PLOS requirements.

**Reviewers’ comments:**

**Reviewer 1:**

*I read this manuscript with interest and found it really relevant and key especially in the context of NCDs. Given the workload experienced by clinicians in SSA in managing patients with chronic diseases, I found the HEP and the role of HEW to be very important work that is being implemented in Ethiopia. I really like the authors effort in Box 1: The context: The Ethiopian health extension program. This work may be useful to the readers of Plos GPH and others actors such as clinicians, researchers or stakeholders working in the field of chronic diseases.*

- Thank you for the positive feedback.

1. *Introduction: Authors can highlight about more on why it’s important to have HEWs in the context of health professional shortage in Ethiopia.*

- We have included sentences highlighting the importance of the HEP in the Ethiopian context. Please see page 3, lines 14 – 15: ‘It was developed to address health system weaknesses, disparities in health outcomes between urban and rural populations, and a critical shortage of skilled health workers’.

2. *The methods section is well presented. I have added few comments on the PDF on areas of improvement.*

- Thank you for the feedback and the comments on the PDF document. We have now revised the manuscript as per the suggestions. Please see page 7, lines 8 – 12; page 7, lines 28 – 30; page 8, lines 1 – 5; and page 9, lines 1 - 3 of the revised manuscript.
- In addition, we have answered the questions raised by the reviewer in the PDF, as shown below.

a. *How practical is this [The PI checked transcription and translation accuracy by transcribing and translating a few interviews independently, page 8, lines 5 – 6]? how many interviews did the PI transcribe himself/herself? was there discrepancy identified?*

- The PI transcribed and translated three interviews and manually compared the content agreement with those completed by the research assistants. Please see page 8, lines 12 – 13: “The PI also checked transcription and translation accuracy by transcribing and translating three interviews”.

b. *Usage of codes may mislead with the coding process.*

- We used NVivo 12 software for data coding. Each participant’s translated interview was saved in a separate document. Participants’ demographic data were also recorded in an excel sheet (called a classification sheet). We have imported the interview documents and the excel sheet into NVivo software together, and this helps to accurately align the code from the interview respondent with the demographic data of the respondent.

c. *As much as the participants are provided with codes and not real names, I feel some of them may be easier to identify from working place column... I suggest deleting the 3rd column and add a column on their other socio-demographics e.g., level of education, age, etc*

- We appreciate the concern about participants’ anonymity. However, we believe having participants’ workplaces in Table 1 provided important contextual information about their roles. Given there are a very large number of such workplaces in the two study regions anonymity is not likely to be compromised when reporting study data.

d. *Be consistent in quotation marks. some places have double Q marks while others single marks.*

- We have used single quotation marks for the quotes included in the text sentences. We haven’t used any quotations for long and standalone quotes. However, we have proofed the whole manuscript for similar typos and other errors.

3. *In the results sections, I was hoping to get more insights on the current roles of HEWs even before talking about their expanded roles after training. I like the way rural and urban areas have been compared and contrasted in the results sections. However, authors can expound more on the similarities and differences.* *For example,* *authors mentioned that UHE-ps were more active or performed more than their rural counterparts. Can the authors explain further why this was the case? For instance, we also know that in urban areas, people are more sensitized than in rural areas, which may make the UHE-ps work easier. In addition, cases of NCDs may be more in urban centres than in rural areas.*

- As described in Box 1 paragraph 1, prior to the introduction of NCDs, HEWs were initially responsible for providing 16 program packages, including family health, disease prevention and control, hygiene, environmental sanitation, first aid, and health education and communication. However, as these other roles were beyond the scope of this study and were mainly drawn from the literature, we have included this information in the background section and not in the results section.

- Regarding UHE-ps, as suggested, we have provided additional information as to why UHE-ps are more active in NCD service delivery than their rural counterparts in the revised manuscripts. Please see page 12, lines 16 – 23: ‘Participants mentioned that UHE-ps took on relatively more NCD roles than their counterparts in rural areas. P. This was because UHE-ps had better skills given all are trained nurses. It may also reflect the fact that NCD packages have been incorporated as part of the urban HEP since the program’s establishment. Other enabling factors include the proximity of the UHE-Ps to health centres and a greater level of NCD awareness among urban residents compared to rural counterparts’.

4. *I like the discussion. However, authors can provide a paragraph on the research and policy implications of their work.*

- Although we have not included a separate paragraph on overall research and policy implications, we have included for each major study finding a corresponding discussion on implications for policy and practice. We have also revised the 1^st^ paragraph of the discussion section to make this more apparent to readers. Please see page 20, lines 23 – 24: ‘Here, we discuss the implications of the findings for policy and practice in Ethiopia and other similar settings’.

5. *Manuscript style and format: My main problem with this manuscript is on punctuation marks. Authors really did poorly in formatting this important work. Punctuation are in many places wrongly placed. Authors use both double and single quotation marks in verbatim, etc. Authors need to read through the manuscript and correct this. See my other comments in the attached PDF.*

- Thank you for the suggestions. We have addressed the comments provided in the PDF document in the revised manuscript. We have also corrected any remaining typographical errors.

**Reviewer 2:**

*This is a well presented and clear analysis. I only have a few minor comments.*

- Thank you for the positive feedback.

1. *The authors state the regions and HEWs were selected purposely. Could the authors please expand on this - what were the factors that informed this selection?*

- We have now explained what factors informed the selection of the two regions and the study participants. Please see page 7, lines 20 – 21: ‘HEWs from the above areas were purposively invited to participate in interviews based on their experience and involvement in delivering the second-generation HEP’.

2. *Could the authors add a box summarising some tentative recommendations based on their findings? Are there any particular lessons that other countries should take note of?*

- We have provided recommendations from the study in the conclusion section. We have also modified some of the sentences in the discussion to reflect the suggestion. Please see the conclusion of the revised manuscript, page 24, lines 22 – 33:

“To address these challenges, we recommend strategies to enhance pre-service training quality, empower HEWs through continuous professional development programs with enhanced NCD content, and strengthen peer-to-peer learning between staff in health posts and the PHC units. A shift to supportive supervision focusing on collective problem-solving, coaching, and mentoring skills is also needed to accompany training reforms. Performance appraisal should also be reformed to establish clear, transparent and achievable targets that reflect the goals of the HEP packages overall, and specifically those related to NCDs.  As some of the limitations on training, supervision and performance also overlap with those of other HEP services, our findings reinforce the view that a robust HEP support system overall would be beneficial for both NCDs and other health conditions”.

3. *Please make use of the COREQ (or equivalent) checklist and include as a supplement -* [*https://www.equator-network.org/reporting-guidelines/coreq/*](https://www.equator-network.org/reporting-guidelines/coreq/)

- Thank you for the suggestion. We have attached the completed COREQ checklist as a supplement file in our resubmission. We have also included reference to COREQ in the methods section of the revised manuscript. Please see page 9, lines 8 – 10.

4. *There were several 'local research assistants'. Should they be included in the authorship?*

- We have followed the International Committee of Medical Journal Editors (ICMJE) and the PLOS Global Public Health authorship criteria for authorship. And we believe the research assistances didn’t meet all four conditions to be listed as authors in the paper, as they were only involved in the data collection process. However, their contributions have been duly acknowledged in the manuscript.

**Reviewer 3**

*This study is very unique in a number of ways; firstly it seeks to appraise and report the extent to which responsiveness to training and supervision of health extension workers (HEW) enhance and support needed skill-building for NCD service delivered by these category of health works in Ethiopia's health extension program (HEP), as suggested by the recommended revised title; "Training and supervision of Health Extension Workers in the delivery of non-communicable disease services in Ethiopia: Outcome of a Qualitative Study". Beyond this, there is the argument that the establishment of the cadre of healthcare workers has rationale underpinning it and is justified. The later argument can only be sustained for policy reform if it is based on sound theoretical and conceptual framework which is absent in the paper. When adequately written, it has policy implications to fully recognize Ethiopia's health extension program and provide the equity it deserves.
However, the following are areas in the manuscript that needs to be strengthened to give it a clear and justifiable argument.*

- Thank you for the positive feedback. We have addressed the comments as shown below.

1. *Title: The title for this study appears inappropriate for a qualitative research design. The use of the word "influence" gives it an intervention profile whereas it is still an observational study. Therefore, a suggestion for modification to align adequately with the design would be; "Training and supervision of Health Extension Workers in the delivery of non-communicable disease services in Ethiopia: Outcome of a Qualitative Study".*

- Thank you for the suggestion. We have now revised the title of the manuscript as follows: ‘Community health extension workers’ training and supervision in Ethiopia: exploring impact and implementation challenges for NCD service delivery”

2. *Abstract:*
   2.1. *The abstract does not adequately represent the study. It lacks information about the problem warranting the study and clear statement of objective, methodology, therefore, as it is expected, the abstract should consist of four distinct paragraphs; it is recommended that the abstract be modified to align adequately.*

- Thank you for the suggestion. Our abstract was prepared following the journal’s format and contained all the required four sections: background, methodology, results, and conclusions. Please kindly see our responses to the remaining suggestions in this section below.
  2.2. *Background: The shortage of skilled health workforce and the rising morbidity and mortality from non-communicable diseases (NCDs) in resource-limited settings, such as Ethiopia and elsewhere, have made community health workers' (CHW) programs an essential strategy to address the growing NCD burden. However, the challenge of a growing disease burden at the primary prevention level requires skill-building responsiveness from the respective health systems. In the context of increasing disease burden, this study sought to appraise and report the extent to which responsiveness to training and supervision of health extension workers (HEW) supported needed skill-building for NCD service delivery in Ethiopia's health extension program (HEP).*
- We greatly appreciate the reviewer’s inputs in the background and method sub-sections of the abstract. As some of this information is already available in subsequent sections, and with intention to provide the reader concise information in the abstract section, we prefer to keep the background as it is.
  2.3. *Methods: A qualitative key-informant in-depth interview (KII) study design was conducted in 2019 in two regions of Ethiopia, the Tigray Region and the South Nations, Nationalities and Peoples Region (SNNPR), and selected 27 consenting policy-makers, district health officials, health center representative and HEW from the Federal Ministry of Health at the federal, regional, district levels who participated. Interview guides were developed guided by the context of the health system responsiveness to the rising NCD burden, recognition of the role of HEW in addressing NCDs, health system strengthening through training and supervision, and principles of primary prevention and modes of intervention..."*
- As in our response 2.2, we are again concerned by word limits and repetitions of details available in the main text. However, we have captured the reviewer’s suggestions in the main method section of the revised manuscript. Please see page 7, line 31 and page 8, line 1.

3. *Background*
   3.1. *It would be observed that the narrative in the background does not specifically set an argument effectively defining the underpinning problem phenomenon. The principles for introducing the HEP is based on Health promotion and disease prevention, which are purely public health principles, thus requires theoretical and conceptual clarifications as part of the review required to establish the scientific foundation.
        The last sentence stating the objective of the study mentions "...vis-a-vis their role in NCD prevention in Ethiopia's HEP." This is premised on the health promotion principles at the primary level of disease prevention and control contextualized in the epidemiological principles of the natural history of disease pathogenesis so that their function as HEW are not confused with the clinical function of the physicians and create a conflict needs to be clearly delineated in the literature defining their relevance. This theoretical and conceptual clarification needs to be included in Box 1 for purposes of establishing clarity of the basis of introducing this aspect of the health workforce, as complementary and not duplication, because these are not clinicians.*

*Another function in the control of NCDs that HEWs may be involved with would be ensuring, by patient education and counselling that the individuals already diagnosed with an NCD and medications prescribed for treatment by qualified medical officer, that they learn to take their medication as prescribed.*

*This the theoretical and conceptual framework considered at this point provides the public health scientific basis for the argument in the study that "HEW serve to facilitate implementation of primary prevention of Non-communicable diseases that follow a defined epidemiological trajectory of the natural history of disease pathogenesis elucidated by symptomatology of NCDs. The prevention functions of the HEWs is essentially within the primary level of prevention with the two strategic modes of interventions, health promotion and specific protection.*

- Thank you for the observation on prevention and HEWs’ role in the context of the overall health system. We agree that the HEWs are not trained to provide clinical care; however, over time, the HEP packages were expanded from solely promotive and preventive services to a more comprehensive package, including basic curative services. We highlighted these facts on page 3, lines 16 – 22, and in Box 1.
  3.2. *The BOX in the background to the study: There is no basis for creating Box 1 rather this would have been part of the major narrative of the background but elucidating the theoretical and conceptual basis of establishing the HEP.*
- Thank you for the suggestion. As highlighted by reviewer 1, we believe the use of the Box in this manuscript is important as it synthesises the key contextual information on the HEP that is pre-requisite knowledge for the study conducted. By putting this information into a box, it helps the background section to be more concise. Readers not familiar with the context can access this information here while those who are well informed about the Ethiopian HEP can gloss over this section.

4. *Methods*
   4.1. *The methodological approach must be consistent with what is stated in the abstract. The study design was "key-informant in-depth qualitative interviews". Reflect this in both the abstract and methodology sub-section of the report.*

- We have revised the language. We now have the phrase “in-depth qualitative study” in both abstract and methods sections.
  4.2. *Under "Participant recruitment and data collection"
       The statement "HEWs from the above areas were purposively invited to participate in interviews." is not complete. It should include the justification for the purposive nature of the selection, ie.* *"HEWs from the above areas were purposively invited to participate in interviews because they are community-based health workers who have experience to share". The basis of purposive sampling is that the sampled have direct relevance to the context and not just any random persons not related to the subject of consideration.*
- We have revised the sentence, and now it reads as follows: ‘HEWs from the above areas were purposively invited to participate in interviews based on their experience and involvement in delivering the second-generation HEP’. Please see page 7, lines 20 – 21.
  4.3. *Developing the interview guide: The statement "Interview guides were developed after reviewing* *different national strategic documents and literature from other settings…" should clarify what specific issues were considered as constituting basis for items of the interview guide. It would have been very appropriate to include the* *theoretical and conceptual framework as the literature basis.
       For emphasis sake; the theoretical and conceptual clarifications constitutes aspects of the literature review contents mentioned above, but this is not obvious in the manuscript at the moment.*
- As we mentioned in the manuscript, we reviewed different national strategic documents and community health workers (CHWs) literature while developing the interview guides. In addition, the qualitative data analysis was informed by the broader CHW performance framework developed by Agarwal et al, 2019, which is one of the comprehensive CHW conceptual frameworks that propose standardised metrics for CHWs. Please see page 8, lines 24 – 27.

5. *Results*
   5.1. *The table 1 should have a modified title "Table 1. Characteristics of study participants". There should also be indication of the gender, length of work experience and possible level of training of participants.*

- We have modified the table title as suggested. In line with our response to the first reviewer’s comments (see #2.c), we think that including additional participant demographic information—on top of their workplace data—may compromise the anonymity of the individuals concerned. In addition, as indicated earlier, capturing the role and functions of the participants are more germane to the study than their demographic profile.
  5.2. *For the results presenting the context, to establish coherence with the public health principles of prevention practices for which this cadres of healthcare workers are established, the recommendation of clarity of framework is necessary. With the theoretical premise elucidated, results should align adequately for consistency and coherence. The context needs minor revision to establish alignment. The first sentence "...the newly revised HEP encompasses the prevention, promotion, early screening of NCDs…". The role of the HEWs is within the primary level of prevention and the corresponding modes of intervention which are health promotion and specific protection. Please refer to the WHO definition of health promotion.*
- We agree with the reviewer’s suggestion to align our results with what is stated under the HEWs’ role. As we noted in response to comment #3, the HEWs’ roles were expanded over time. For instance, according to the revitalised package HEWs were mandated to provide health education on NCDs and their risk factors, undertake screening for early NCDs detection, and refer patients with more complex needs to health centers (please see page 3, lines 16 – 22). However, our qualitative findings revealed that their role was currently limited to NCD prevention services (page 11, lines 5 – 13).
  5.3. *We shall note here that health promotion has three components of Health Education which these HEW are trained to perform, Service strengthening trough training and Policy advocacy. It would be safe to define the limits of function. The results having the memorable quotes illustrating lessons learned are appropriate.*
- Thank you for the feedback.

6. *Discussion*
   6.1. *It would have been very appropriate within the first paragraph of the discussion section to reiterate the objective of the study.*

- We have now incorporated the objective of the study into the discussion. Please see page 20, lines 19 – 21.

6.2. *The second paragraph begins with "Training for NCDs lacked breadth and depth -it was …" this is not clear. Whose training is being mentioned here?*
- We have revised the sentence to give more clarity. The sentence read as: “NCD training for HEWs lacked breadth and depth…” in the revised manuscript. Please see page 20, line 25.
  6.3. *In paragraph three the statement "The limitations we observed in HEW training for NCDs are similar to findings from a recent government report that acknowledges that HEWs continue to have suboptimal knowledge and skill to provide other HEP services…" did not provide reasons for the observations. This is very important, and omission of a reason weakens the argument in the study.*
- We have now included the information as suggested in the revised manuscript. Please see page 21, lines 5 – 9.
  6.4. *The conclusions should have considered policy statement regarding the establishment of the HEP to establish consistency is aspects considered in the study or lack of it.*
- Thank you for the feedback. Please see our reply to reviewer 1: #4.

Thank you again for your feedback and for considering our revised paper. Please do not hesitate to contact me if you have any further questions.

Best regards,

Azeb Gebresilassie Tesema
